# Editorial Notes

**Published:** 1902-06

**Authors:** 


### Editorial Notes.

**Edward Long Fox Memorial.**—Many of our subscribers will be glad to have an opportunity of showing their regard for our late friend and colleague, Edward Long Fox, by contributing towards this desirable object. At a meeting of friends recently held in the Chapter Room of the Bristol Cathedral, the Right Hon. Lewis Fry, P.C., took the chair. He regretted that the Dean was prevented from being present as he had intended. It was resolved that the form of the memorial should be—(1) a brass tablet to be placed in the Cathedral; (2) the establishment of an Annnal Lecture on some subject connected with medical science, to be known as the Long Fox Lecture, to be delivered at the University College; (3) that any further available funds be devoted to the assistance of a student who would become a medical missionary. Donations may be sent to the Treasurer, the Rt. Hon. Lewis Fry, Esq., P.C., Bristol Old Bank, Clifton, or to either of the Hon. Secretaries, W. W. Asquith, Esq., 34 College Road, and Dr. Markham Skerritt, Edgecombe Villa, Clifton.

**The Crusade against Tuberculosis** goes on slowly. The National Association continues to grow in activity and influence, and there is a general awakening in the country as to the need for the provision of sanatoria for the poor. "There are still many cities and towns in Great Britain where the municipal authorities pass by on the other side, and leave the poor consumptive to the casual Samaritan—or to death in a ditch." (*Practitioner*, April, 1902, p. 375). For these Professor Osler has recently addressed the municipality of Baltimore, and he adds: "If you can get the people to awake, it dosen't make any difference if the mayor and city council are asleep." Here, in Bristol, and in the neighbouring city of Bath the reverse is the case; the councils are fully alive to the necessity for decided action, but the active philanthropy of the public has not yet risen to the occasion.

The Bath Council voted £500 on the building being completed and £120 annually for the maintenance of two beds. At a meeting of the Bristol Health Committee it was resolved: "That on completion of the building and equipment of the proposed sanatorium for consumptives at Winsley, the sum of £1000 be voted to the Gloucester, Somerset, and Wilts branch of the National Association for the Prevention of Consumption and other forms of Tuberculosis, and the sum of £260 be paid annually for the exclusive use of four beds for Bristol cases, provided (1) that one of the trustees in whom the sanatorium is to be vested be a member of the Town Council of Bristol; (2) that four members of the Council be on the Executive Committee." This recommendation has now been adopted and passed by the Town Council.

The **discussion on Plague**, opened by Dr. Edward Klein, at the meeting of the Bristol Medico-Chirurgical Society, will have directed attention to a subject of which we in this country know little, and do not commonly give ourselves the trouble to learn. When we realise what this disease is in India, it becomes obvious that such apathy is almost criminal. The importance of the plague question, looking alone at the mortality in India, is enormous. In the week ending March 1st there were 21,789 deaths, in the previous week there were 14,946, whereas in the corresponding week of 1901 the number was only 6,991. In Bombay city alone 750 died of plague in one week (*British Medical Journal*, March 29th, 1902, p. 786). It is a disease worth keeping out.

**Nursing Experiences on the "Hospital Ships" at Dartford.**—The following thrilling and pathetic account of some nursing experiences of small-pox are from the pen of a former sister at the Royal Infirmary, Bristol. It tends to show that it is worth while to prevent such a disease as is depicted, and should do something towards the suppression of the anti-vaccination faddist. The more widely such knowledge is disseminated the more eager will these persons be to give themselves and the world that protection which vaccination alone can give.

"Thrice in 24 hours patients are brought from London by steamer, and, weather being favourable, two hours at most should see them on board. Fog disarranges everything, the steamers can neither leave the wharf nor the ship, and the consequent accumulation of patients first on the wharf and then on the ship is very trying. All clothes are removed at the wharf, disinfected, and returned to their owners when leaving Gore Farm. The patients are wrapped in warm flannels and blankets for the outer journey, and are accompanied by a nursing staff; they are carried to the wards on stretchers or in chairs by a small army of porters, and the resident medical officer at once examines and classifies the scars, rash, &c. . . . On the ships the patients are on feather beds (at "the Extension" and different new buildings I hear a specially prepared wool mattress is being tried). Bad cases cannot stand having their beds made, so are lifted on to fresh clean ones, and with some this is necessary several times during the day or night. Faces are so disfigured and change so rapidly that it is not always easy to identify them. We have an unlimited supply of feather pillows, sheets, handkerchiefs, &c., and patients always look absolutely clean. Eyes, mouths, and throats are done every four hours or more frequently: the tongues and throats of confluent cases are ulcerated and in a dreadful condition: the hœmorrhagic ones are almost hopeless to deal with, as the bleeding is increased by the gentlest touch. Bed sores have to be looked for, as they come quickly and slough deeply. Spirit cannot be used and powder cannot be rubbed on as preventatives, but the parts are dusted from large tins with perforated lids. I don't think I have seen one bed sore heal. Every patient is blanket washed twice in twenty-four hours, using plenty of soap, and not rubbing but soaking the skin and carefully drying. This gives great relief, and diminishes the small-pox smell. Cold is carefully guarded against; a sleeveless flannel shirt is first put on, over this a thick cotton one, both open down the back. . . . The temperatures are not often over 103°, the average is about 100°. To give sufficient nourishment is a great part of small-pox nursing, and until death is imminent—or the patient imagines he is being poisoned—is not difficult. Thirst is great, and many pints of milk are easily disposed of. . . . Brandy is the only stimulant I have been ordered to give, in small quantities and often; two or three teaspoonfuls every two hours as a rule. . . . For the faces every sort of treatment has been tried. In the early days of the epidemic a mask of Whitehead's varnish was the correct thing, but proved disappointing. Ointment masks and fomentations are popular; but nothing seems to prevent scarring. . . . The soles of the feet are often very painful, and are fomented; the snipping of the blisters there and on the limbs I cannot describe: some patients are in fomentations from head to foot.

Delirium is the worst part of small-pox nursing; it comes on so suddenly, and sometimes when least expected; at your first round you may attend to the face of a civil and pleasant man, and at your next visit to him you find yourself looking into the eyes of a madman who is inviting you to come with him through the window and find a resting place in the river. . . . Suicide is the one idea, and their whole mind is bent on it, and as they are extremely cunning and rapid in their movements, men and women alike are a dreadful responsibility. A month ago one of these maniacal cases escaped through a small window in the roof and drowned himself in the river.

The appearance of the delirious cases is peculiar; frequently one arm is raised and trembling, and the index finger appears to be pointing at something high up. . . . The hœmorrhagic cases are the saddest; from the first they are considered hopeless, although they do not always look bad on admission. They are conscious to the end, and their death is terrible. In other hœmorrhagic cases there is a small red rash, the face swells past recognition and becomes almost black; these suffer much from the mouth and throat. . . . In having charge of the ward for delirious patients I may consider myself fortunate, as it enabled me to nurse small-pox in its worst and most malignant forms. . . . An hour spent in that particular ward would, I am sure, have cleared up every doubt as to the blessings of vaccination. . . . Nothing seemed to make them sleep or quiet them—opium, bromide, trional, paraldehyde, were all no good. Very large hypodermics of morphia answered, but the risk of collapse had to be considered. . . . I shall always associate with the ships the tramp, tramp, of the stretcher bearers carrying the dead from the wards; it went on all night; it got on the nerves, especially when a patient buried his head in your apron and implored you not to let them come for him next.

At any hour of the night the Roman Catholic priest from Dartford would appear silently at my side—and having attended to some dying man—as silently depart; but, out of the tail of my eye I have seen him give drinks, shake pillows, and leave comfort in many ways behind him.

Some, who have not tried it, think there is little nursing possible in small-pox, but I cannot agree with them . . . And when one poor dying thing took my hands in his and felt and rubbed them for a time, and saying "Like mother's hands," bent and kissed them, my time here did not seem altogether wasted. . . . The complete wipe out of families is astonishing; it is a common thing to have the husband, wife and many children here. . . . One nurse only has taken small-pox here. . . ."

